# Endoscopic-assisted transorbital surgery: Where do we stand on the scott’s parabola? personal considerations after a 10-year experience

**DOI:** 10.3389/fonc.2022.937818

**Published:** 2022-07-15

**Authors:** Iacopo Dallan, Lodovica Cristofani-Mencacci, Giacomo Fiacchini, Mario Turri-Zanoni, Wouter van Furth, Matteo de Notaris, Miriana Picariello, Enrico Alexandre, Christos Georgalas, Luca Bruschini

**Affiliations:** ^1^ Ear Nose Throat (ENT) Audiology and Phoniatric Unit, Department of Surgical Pathology, Medical, Molecular and Critical Area, AziendaOspedaliero-UniversitariaPisana, University of Pisa, Pisa, Italy; ^2^ ENT Unit, Department of Biotechnology and Life Sciences, Ospedale di Circolo e Fondazione Macchi, University of Insubria, Varese, Italy; ^3^ Neurosurgery Unit, Department of Ophthalmology, Leiden University Medical Center, Leiden, Netherlands; ^4^ Neurosurgery Operative Unit, Department of Neuroscience, “San Pio” Hospital, Benevento, Italy; ^5^ Medical School, University of Nicosia, Athens, Greece

**Keywords:** transorbital endoscopic surgery, orbital surgery, multiportal surgery, TOAs, learning curve, skull base surgery

## Abstract

Transorbital approaches are genuinely versatile surgical routes which show interesting potentials in skull base surgery. Given their “new” trajectory, they can be a very useful adjunct to traditional routes, even being a valid alternative to them in some cases, and add valuable opportunities in selected patients. Indications are constantly expanding, and currently include selected intraorbital, skull base and even intra-axial lesions, both benign and malignant. Given their relatively recent development and thus unfamiliarity among the skull base community, achieving adequate proficiency needs not only a personalized training and knowledge but also, above all, an adequate case volume and a dedicated setting. Current, but mostly future, applications should be selected by genetic, omics and biological features and applied in the context of a truly multidisciplinary environment.

## Introduction

The term “transorbital approaches” (TOAs) describes a very wide and heterogenous group of procedures. They all share one basic aspect, which is: the procedure is performed passing through the orbital space. The use of endoscopes as visualization tools introduces the concept of endoscopic-assisted transorbital surgery. Many transorbital routes are now available and, since their introduction in 2010 by Kris Moe (1), this “new” philosophy has gained increasing popularity. Initially described as ancillary alternatives to traditional routes, TOAs have evolved to the state of well-established surgical procedures, acting as valid alternative to traditional approaches for selected lesions (2). As evidence of the latter fact, when searching in Pubmed the terms “transorbital endoscopic”, the results show a consistent increase in publications in recent years, witnessing a growing and outstanding interest in this topic. In this paper, after reviewing current literature and retrospectively reviewing our 10-years experience in transorbital surgery, we present some considerations on three main aspects: actual possibilities, learning curve processes and future developments. As for any other surgical approach, also for TOAs there are pros and cons as there are good and bad indications, although their clear understanding is actually far from complete.

## Actual possibilities

**Figure 1:**
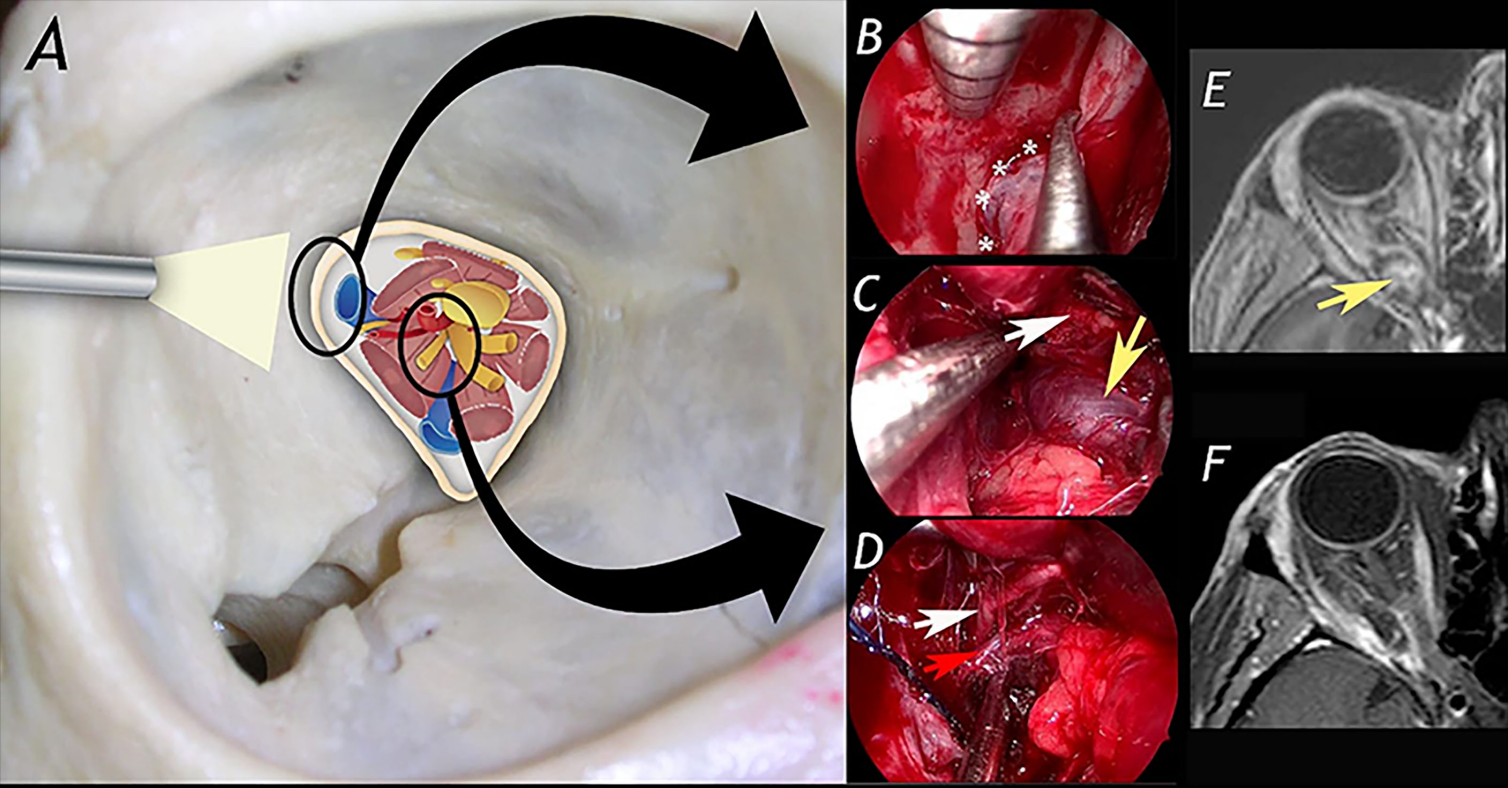
A 42-year-old woman affected by right orbital apex cavernous hemangioma (with progressive worsening of right visual field) was treated via superior eyelid endoscopic-assisted approach with complete resection of the lesion and no significant morbidity. **(A)** schematic drawing showing anatomical structures in the orbital apex. In **(B–D)** surgical steps of the procedure. **(B)**: exposure of the lateral aspect of the superior orbital fissure. **(C)**: identification of the cavernous hemangioma. **(D)**: dissection of the lesion from superior division of III cranial nerve and ophthalmic artery. **(E, F)** show pre- and post-operative MRI. White asterisks: lateralaspect of superior orbital fissure; white arrow: superior division of the oculomotor nerve; yellow arrow: cavernous hemangioma; red arrow: ophthalmic artery.

Since its first description (3), the use of endoscopes in orbital surgery has gained, with years, tremendous interest. In recent years TOAs have been used for the treatment of pathologies located within the orbit or adjacent to it (**Figure 1**) (1, 4–9) or even to target distant areas using the orbit as a corridor (10–21). Moreover, in the contest of multidisciplinary and modern surgery, TOAs can be performed as a single procedure or be part of a multiportal surgery. It is well established that selected patients can benefit from the potentially better exposure provided by a combination of approaches. Furthermore, as recent literature reveals, even intra-axial lesions of the temporal lobe have been managed *via* TOAs (21) and pre-clinical studies on neurovascular surgery have been conducted (22). From a surgical point of view, transorbital approaches have been demonstrated to offer equivalent exposure to traditional routes (23, 24), allowing a safe and effective management of selected lesions. Thus said, like any other approach, TOAs are obviously not suitable for all cases, and present its own pros and cons (**Table 1**). But certainly the orbit can be considered as a reliable port to overcome intrinsic limits of traditional routes, both endoscopic-assisted and not. Obviously TOAs present some intrinsic limitations. Among these, the possible compression of the orbital content necessary to gain adequate room for working; the need for a very careful management of eye surface and the not easy control in case of major bleeding (although this last concern is not typical of transorbital approaches rather than of endoscopic-assisted approaches). A recent systematic review reported a very wide range of pathologies managed *via* TOAs (2). The very large majority of this cohort are represented by spheno-orbital meningiomas, meckels’ cave schwannomas, inflammation/infection and csf leaks. In this respect, the long-term experience of the authors confirms these data. In **Table 2** personal data and other groups’ experiences are summarized.

**Table 1:** Advantages and limits of TOEA and the most commonly used transcranial routes.

	ADVANTAGES	LIMITS
***Transorbital***	• *Short distance to the target.* • *Very limited bony work, good cosmesis.* • *No nerve or major vessel crossing* • *Good control of lateral aspects of superior orbital fissure and cavernous sinus.* • *No need for brain retraction.* • *Control of upper aspects of infratemporal fossa, as well control of anterior pole of the temporal lobe and orbital part of the frontal lobe.*	• *Temporal fossa invasion* • *Lesions extending too posteriorly (squama temporalis)* • *Major vessels encasement*
***Supraorbital***	• *Small incision* • *Great control of anterior cranial base and supra- and parasellar region (superior aspect)*	• *No control of the anterior temporal lobe region and of the infero-lateral aspect of the cavernous sinus (inferior parasellar region)* • *No control of the infratemporal fossa region.*
***Fronto-temporal***	• *Good exposure of the anterior and middle cranial base*	• *Need for bony work and large skin flap.* • *Very difficult control of the infratemporal fossa regions.* • *Need to manage the anterior part of the temporal lobe to get the lateral wall of the cavernous sinus (parasellar region).* • *Frequent need for brain retraction*
***Pterional***	• *Good exposure of the anterior and middle cranial base.* • *Good exposure of the Sylvian fissure*	• *Need for bony work and large skin flap.* • *Difficult control of the infratemporal fossa regions.* • *Need to manage the anterior part of the temporal lobe to get the lateral wall of the cavernous sinus (parasellar region)*
***Subtemporal***	• *Good exposure of the lateral aspect of the parasellar region, anterior pole of the temporal lobe and antero-lateral posterior fossa*	• *No control of the anterior cranial base* • *Need for bony work and large skin flap.* • *Need to manage the anterior pole of the temporal lobe to get space for lateral aspect of parasellar region.* • *Need for retraction for suprasellar access.* • *Very difficult control of infratemporal fossa regions.*
***FTOZ and variations***	• *Wide exposure of anterior and middle cranial base.* • *Good exposure of the supero-lateral aspect of the orbit.* • *Possibility to reach even the interpeduncolar and prepontine cisterns*	• *Need for extensive bony work and large skin flap.* • *Difficult control of infratemporal fossa regions*

**Table 2:** Personal published and unpublished data and other groups'experiences with TOAs.

	Intra-orbital lesions	Skull Base lesions	Intra-axial lesions
Our published cases	14 (8, 9, 25, 26)	90 (16, 27)	
Our unpublished cases	23	113	1
Jeon C	10 (28)	6 (29)	3 (29)
Almeida JP		2 (30)	
Park HH		12 (31)	7 (21)
Kong DS		23 (7, 32)	7 (32)
Others		48 (33–35)	21 (33)

## Learning curve item

**Figure 2:**
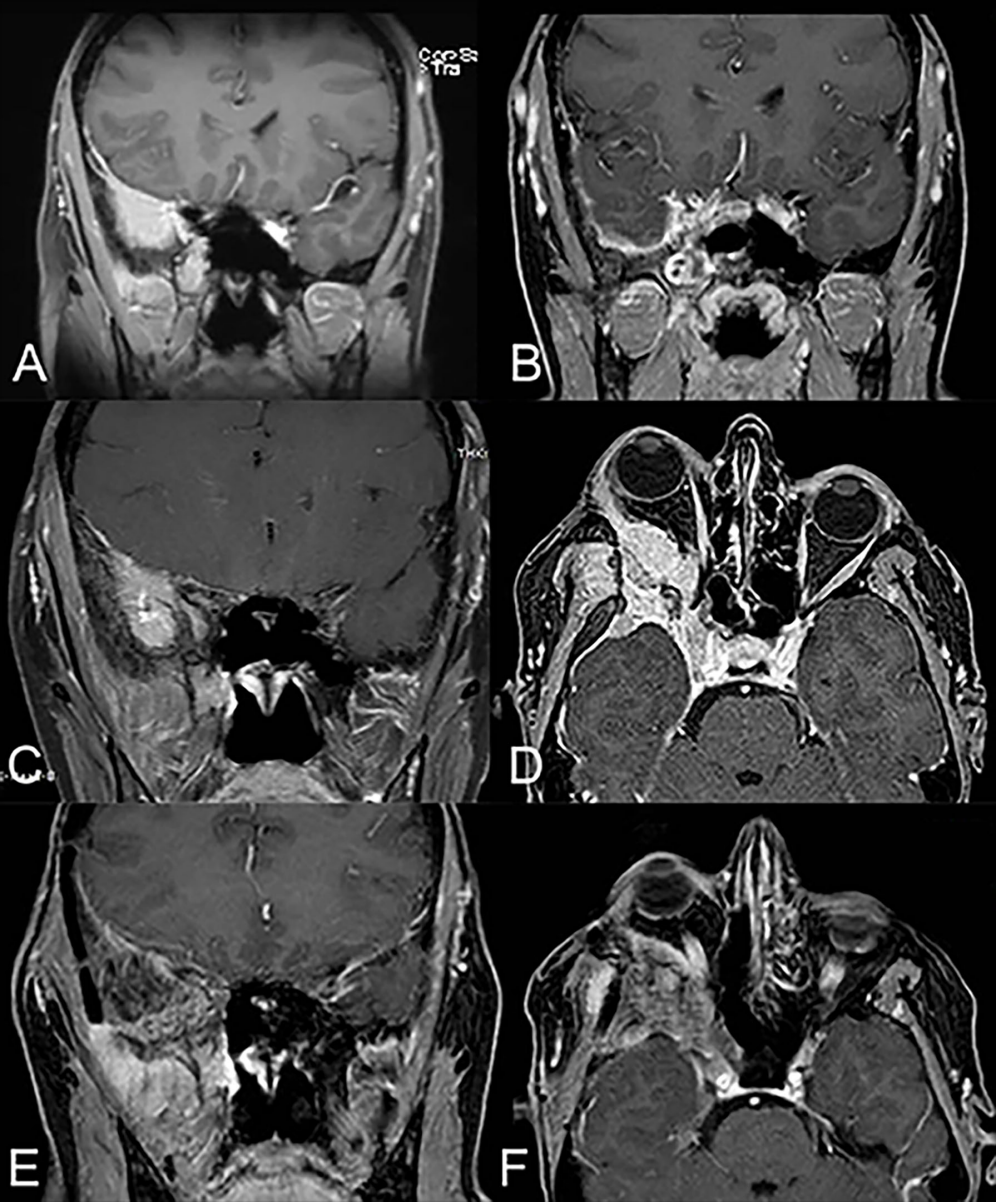
A 48-year-old woman affected by right spheno-temporal meningioma (pre-operative MRI depicted in **(A)**) was submitted to endoscopic-assisted resection *via* combined trans-nasal and trans-orbital corridors, obtaining gross total resection (post-operative MRI depicted in **(B)**). During the follow-up, 5 years after the primary treatment, she developed right proptosis and periorbital pain and the MRI documented a recurrence of the meningioma (MRI in panel **(C)** involving also the transorbital corridor (MRI in panel **(D)**. The patient was then submitted to revision surgery *via* transcranial approach (right frontotemporal orbitozygomatic craniotomy). The MRI performed two years after revision surgery were clear from macroscopic recurrence of disease **(E, F)**, with partial resolution of the right orbital pain and proptosis of the patient.

Generally speaking, surgeons spend most of their professional life acquiring new surgical skills and learning new surgical procedures, the real value of which will be judged by time. As a matter of fact, understanding the surgical anatomy of TOAs requires a certain eclecticism and dedicated training. It is well established that, when learning a new procedure, performance tends to improve with experience. Graphically plotting performance against experience produces what is called a “learning curve” (36). This model applies across the full spectrum of medical science and procedures; however, with the advent of technically demanding minimally invasive techniques, surgery in particular is where there are specific and potentially dramatic implications. As demonstrated in colorectal cancer surgery, surgical experience and case-volume, combined with technological resources, are good prognostic factors for the patients’ outcome. Therefore, before reaching an adequate proficiency (which means *being skilled in doing or using something*), several obstacles have to be overcome. The most important of these obstacles is probably the volume of cases. In other words, the number of cases performing a specific procedure seems to be critical (37). The real problem is that this number is not known in skull base surgery. As well underlined by Snyderman, the learning curve in endoscopic skull base surgery has to deal with issues of knowledge of endoscopic anatomy, quality of instrumentation, 2-D visualization and team dynamics (37). Furthermore, the quality of the learning experience is of paramount importance. Different situations offer different information to the surgeon. All these factors can be important in determining the final outcome (than means a proper management of the patient). Historical data seem to confirm that, in respect to pituitary surgery, proficiency can be achieved after 20-50 cases (38–41). But these numbers do not consider a lot of factors. If we transpose these considerations to TOAs we easily understand that several concerns can be raised. First, as TOAs are a “recent” approach, there are a lot of controversies regarding their correct indications. An honest rethinking of our 10-year experience makes us perfect witnesses to this aspect. In all honesty and with our actual experience and knowledge, looking retrospectively we would have not managed with these approaches some of our early-experience patients (**Figure 2**). Unfortunately we consider this a price to be paid in the early phase of any given procedure/treatment. On the other hand, the low number of possible candidates to TOAs procedures and the validity of the coded traditional approaches, make the patient-selection process difficult. In detail, if we consider as possible candidates for TOAs intraorbital pathologies and skull base lesions (mostly spheno-orbital meningiomas and meckels’ cave tumors), we can only collect a small cohort of patients. From an epidemiological point of view, these pathologies are extremely rare. Estimated incidence of sphenoid ridge meningioma (SOM) is about 1, 5 cases per 100000 person-years (42). Trigeminal schwannomas account for less than 0.4% of intracranial tumors. And obviously, not all these cases can eventually be considered good candidates for a TOA. Indeed, if we change perspective and check current existing medical literature, our doubts are confirmed (31, 43–49). Case series on SOM are normally collected over decades and seldom include more than 50 patients. Numbers of trigeminal schwannomas are even less. Furthermore, current literature includes patient series often covering multiple decades, while surgical techniques have improved over the years. These numbers open a serious debate: how can we deal with a proper learning curve in TOAs given the number of cases available? Which is the number of cases to be performed before achieving technical proficiency? From our point of view, confidence with these approaches can be achieved after at least 50 cases (although we have no clear and objective data, our experience of more than 200 TOAs seems to be a sound base for balanced considerations). Obviously, this is only our feeling. Furthermore, we do feel that this proficiency can be maintained only if at least 20 cases a year are performed.

## Future directions

It’s clear that the evolution of surgery, and, in this contest, of TOAs, is still underway. But to what end? On one hand surgeons and hospitals are committed to deliver to the patient always the best possible outcome. But, on the other one, is surgery necessarily the right approach? Everyone talking about future applications of any given or medical procedure needs to be open-minded and, to some extent, provocative. In this respect, we strongly feel that, to best serve our patients, surgeons need to reevaluate their isolated position within the medical profession. If surgeons continue to work within an isolated medical arena, they may risk missing break-event moments and fundamental knowledge. Everyone knows that falling in love with any given procedure, mastering it and getting popularity from it, is very easy. As Ulysses, we, as surgeons, need to be tied to the boat and think of our targets, in order not to surrender to sirens’ voices. For decades and decades surgeons had had a surgery-based approach. In recent years something has changed. Despite this, there’s still a long way to go. Understanding biological, psychological and economical aspects and not limiting our perspective only to surgery will be a game changer. To do this we really need to act, synergistically, in a truly multidisciplinary environment. This multidisciplinary and disease–centered approach will facilitate the development of novel techniques. This will be easier to achieve if both surgical and nonsurgical specialists are involved in the global-procedural management of the patient. In other words, disease-based practices will facilitate a focus on outcomes and patient necessities. In this respect technological evolutions and refinements, such as integrated suites with all facilities available, are a step forward. There is no doubt that surgeons are still missing technologies and real-time clinical data to improve decision-making processes. These are critical in the setting of high-pressure and highly variable situations which happen constantly during any skull base surgery. More specifically, in transorbital procedures, sophisticated autostatic orbital retractors, real-time visual function checking systems and dedicated instrumentations able to increase the identification and dissections of noble structures (e.g. small vessels and nerves) will increase the safety and consequently the efficacy of this kind of procedures. Furthermore, pre-operative functional studies will greatly help in the correct indication of the patients. But only targeted therapies, that imply a truly personalized treatment, based on genetics, omics, and so on, will lead the way to the future.

## Conclusion

Whether TOAs will get or not a sound value for patients is still a matter of discussion. Certainly this “corridor” has expanded the armamentarium of skull base surgeons. The rapidly growing number of publications on this topic reflects a vivid interest. But similarly to what happened in the gold rush, not all the participants can be lucky and not all that shines is gold.

One thing is certain: surgery will not stay the same. As in the 1900s, technology and knowledge will catch up with imagination and the evolution of surgery and medicine will continue. The real revolution will be moving from a surgical-based approach to a truly disease-based approach. Because that’s the worthy goal: to deliver consistently superior patient outcomes regardless of surgeon skills, training or location.

## Data availability statement

The original contributions presented in the study are included in the article/supplementary material. Further inquiries can be directed to the corresponding author.

## Author contributions

ID made substantial contributions to conception and design. He participated in drafting the article and revising it critically for important intellectual content. LC-M made substantial contributions to acquisition of data and analysis; participated in drafting the article and revising it critically for important intellectual content. MP made substantial contributions and participated in revising the article critically for important intellectual content. GF made substantial contributions and participated in revising the article critically for important intellectual content. MT-Z participated in revising the article critically for important intellectual content and he gave final approval of the version to be submitted. WF participated in revising the article critically for important intellectual content and he gave final approval of the version to be submitted. MN participated in revising the article critically for important content and he gave final approval of the version to be submitted. CG-H participated in revising the article critically for important content. He participated in drafting the article and he gave final approval of the version to be submitted. LB made substantial contributions to conception and design. He participated in drafting the article and gave final approval of the version to be submitted. All authors contributed to the article and approved the submitted version.

## Conflict of interest

The authors declare that the research was conducted in the absence of any commercial or financial relationships that could be construed as a potential conflict of interest.

## Publisher’s note

All claims expressed in this article are solely those of the authors and do not necessarily represent those of their affiliated organizations, or those of the publisher, the editors and the reviewers. Any product that may be evaluated in this article, or claim that may be made by its manufacturer, is not guaranteed or endorsed by the publisher.
